# Successful response to the combination of immunotherapy and chemotherapy in cholangiocarcinoma with high tumour mutational burden and PD-L1 expression: a case report

**DOI:** 10.1186/s12885-018-5021-2

**Published:** 2018-11-12

**Authors:** Haibo Mou, Lanfang Yu, Qin Liao, Xuehua Hou, Yinfang Wu, Qiang Cui, Na Yan, Ruobing Ma, Lingjian Wang, Ming Yao, Kai Wang

**Affiliations:** 1 Department of Medical Oncology, Shulan (Hangzhou) Hospital, #848 Dongxin Road, Hangzhou, Zhejiang Province 310022 People’s Republic of China; 2 OrigiMed, 115 Xinjun Huan Road, Minhang District, Shanghai, 201114 China

**Keywords:** Immunotherapy, Next generation sequencing, TMB-H, PD-L1

## Abstract

**Background:**

Cholangiocarcinoma, or bile duct cancer, is a gastrointestinal cancer with limited therapeutic options and a poor outcome. Studies have revealed that some major driver genes are associated with cholangiocarcinoma, but no targeted therapies have been approved. Immune checkpoint inhibitors, which are represented by inhibitors of programmed cell death 1 (PD-1)/programmed death-ligand 1 (PD-L1), have emerged as a potential therapy for multiple types of solid cancers.

**Case presentation:**

A 53-year-old female presented with postoperative recurrence of PD-L1-positive intrahepatic cholangiocarcinoma with a high tumour mutational burden. This patient exhibited a marked response to the combination of anti-PD-1 immunotherapy and chemotherapy.

**Conclusions:**

As far as we know, this is the first case report on the success of the combination of immunotherapy and chemotherapy for advanced cholangiocarcinoma with PD-L1 positivity and a high tumour mutational burden.

## Background

According to the 2010 WHO guidelines, cholangiocarcinoma is stratified anatomically into intrahepatic cholangiocarcinoma (iCCA) and extrahepatic cholangiocarcinoma (ECCA) [1], both of which have a poor prognosis. Surgery remains the primary curative treatment option for biliary tract cancers, but more than two-thirds of patients are not candidates for surgery at diagnosis [2]. Unlike other carcinomas, no targeted agents have been approved for advanced cholangiocarcinoma, and only limited chemotherapeutic options are recommended by the NCCN for patients who are not candidates for surgery [3]. In addition to distant metastasis, most patients with multiple liver lesions and distant lymph node metastasis, other than hepatic hilar lymph node metastasis, are not suitable for surgical treatment. Cholangiocarcinoma is a rare tumour type in Europe and the United States, but it is not rare in China, as the incidence rate is approximately 7 per 1 million people [3]. However, approximately only 20 clinical studies of cholangiocarcinoma drugs have been conducted or are currently underway in China [4]. Most patients are unable to be enrolled in clinical studies. The 5-year overall survival rates for intrahepatic, distal, and peri-hilar cholangiocarcinoma are 22 to 44%, 27 to 37%, and 11 to 41%, respectively [4]. iCCA, which is a malignant adenocarcinoma that originates from the secondary bile duct and its epithelial branches, accounts for 10 to 15% of primary liver neoplasms [5]. The incidence of iCCA has been increasing in recent years. Patients diagnosed with iCCAs may present with atypical clinical symptoms, and thus they will be diagnosed at an advanced stage of disease when surgical resection is not an option. Although iCCA is a rare cancer, the incidence has increased by 165% (from 0.32/100,000 to 0.85/100,000) [5].

Recently, immune checkpoint inhibitors that target PD-1/PD-L1 have facilitated a significant breakthrough in the treatment of multiple solid cancers [6–8]. Compared with the overall survival of patients who are treated with small molecule targeted drugs, conventional chemotherapy drugs and PD-1/PD-L1 inhibitors can lead to significant improvements in OS and PFS in some cancers [9–12]. Potential indicators that can predict response to treatment include PD-L1 expression, tumour mutational burden, microsatellite instability (MSI), and mismatch repair deficiency (dMMR) [13–15]. Different studies have reported the conflicting predictive role of PD-L1 expression in the response to anti-PD-1/PD-L1 therapies in patients with different tumour types. The FDA has approved pembrolizumab as a first-line therapy for advanced non-small cell lung cancers (NSCLCs) that have high PD-L1 expression and no driver mutations (tumour proportion score (TPS) ≥ 50%) [16]. Herein, we reported a patient who was diagnosed with an intrahepatic cholangiocarcinoma with a high tumour mutational burden (TMB) and high expression of PD-L1 (TPS reached 80%). This patient achieved complete remission (CR) and experienced fewer side effects after treatment with pembrolizumab and chemotherapy.

## Case presentation

**Fig. 1:**
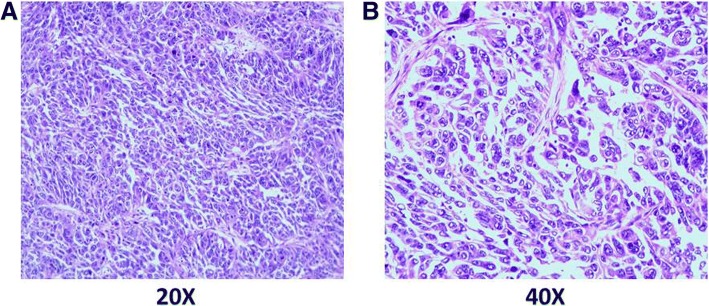
Histology of the tumour tissue: **a** 20X and **b** 40X

A 53-year-old female presented with intermittent abdominal distension and was admitted to the hospital. The patient had a history of chronic hepatitis B infection, and as a result, received antiviral therapy. No scleral icterus or xanthochromia was detected, Murphy’s sign was negative, and the patient’s performance status score was 1. Abnormal prothrombin, carcinoembryonic antigen (CEA) and alpha-fetoprotein (AFP) levels were within the normal range; however, cancer antigen 19–9 (CA19–9) level was increased to 66.81 U/ml, respectively. Magnetic resonance imaging (MRI) revealed a space-occupying lesion in the left liver, while no abnormal space-occupying lesions were found in the lungs, breast, gastrointestinal tract, or other areas that are prone to liver metastasis. Therefore, this lesion was considered a primary liver tumour. The patient underwent a curative resection in August 2016. The tumour tissues were sent for pathological evaluation, which indicated poorly differentiated adenocarcinoma. The tumour was 8*5.5*9.5 cm in size and was also necrotic and nodular with vessel invasion (Fig. 1a and b); however, invasion of the nervous system or surgical margins was not observed. An immunohistochemical analysis revealed the following: AFP(−), CA-125(−), CD10(−), CD34(blood vessel+), CKpan(+), CK7(−), CK19(+), CK20(−), HCV(−), HBcAg(−), HBsAg(liver+) Ki-67(50%+), P53(90%+), TTF-1(−), vimentin(+), WT1(−), and Gly3(−). These results suggested a diagnosis of stage IIIB iCCA (pT2N1M0).

**Fig. 2:**
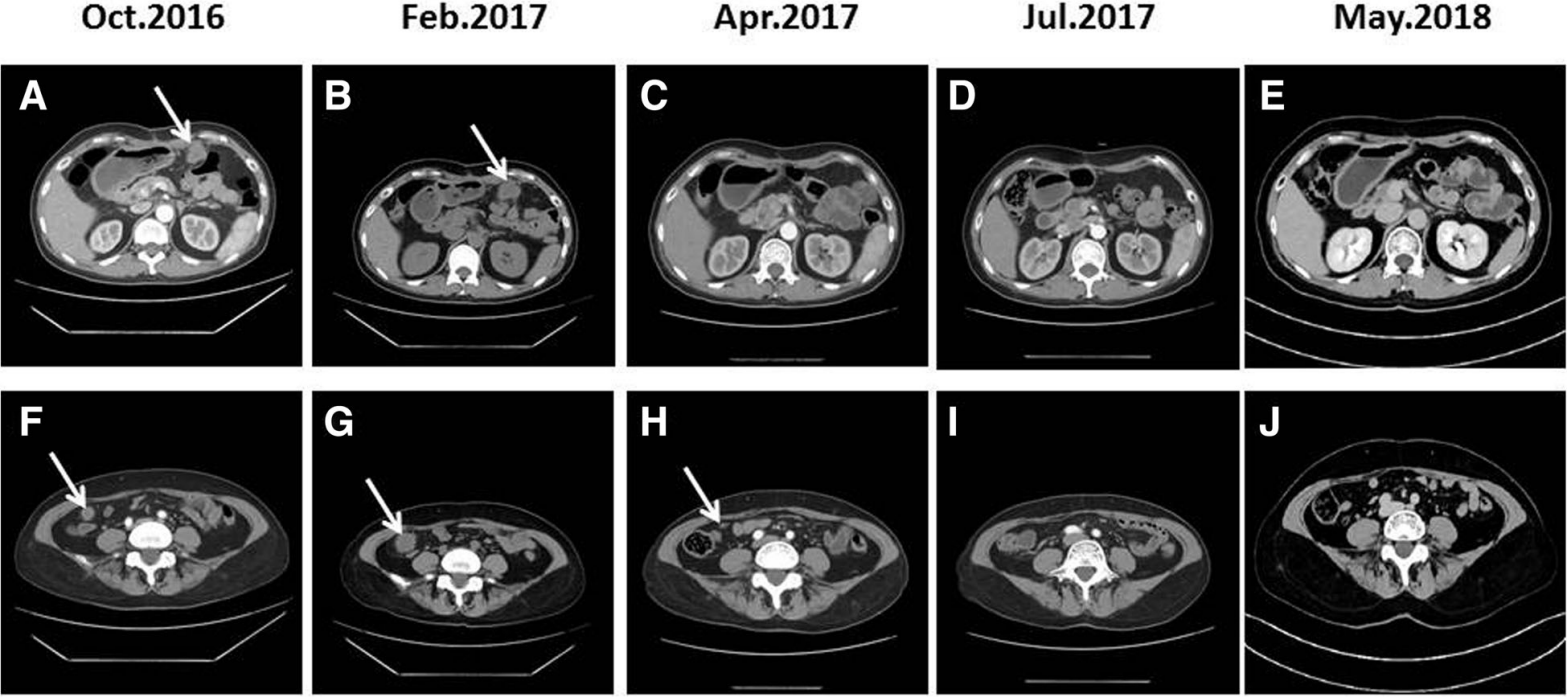
Computed tomography scan shows the different stages of the metastatic lesions in the coelom over time (white arrows). **a**-**e** indicate the lesion located in the upper left omentum majus and **f**-**j** indicate the lesion located in the lower right peritoneum

**Fig. 3:**
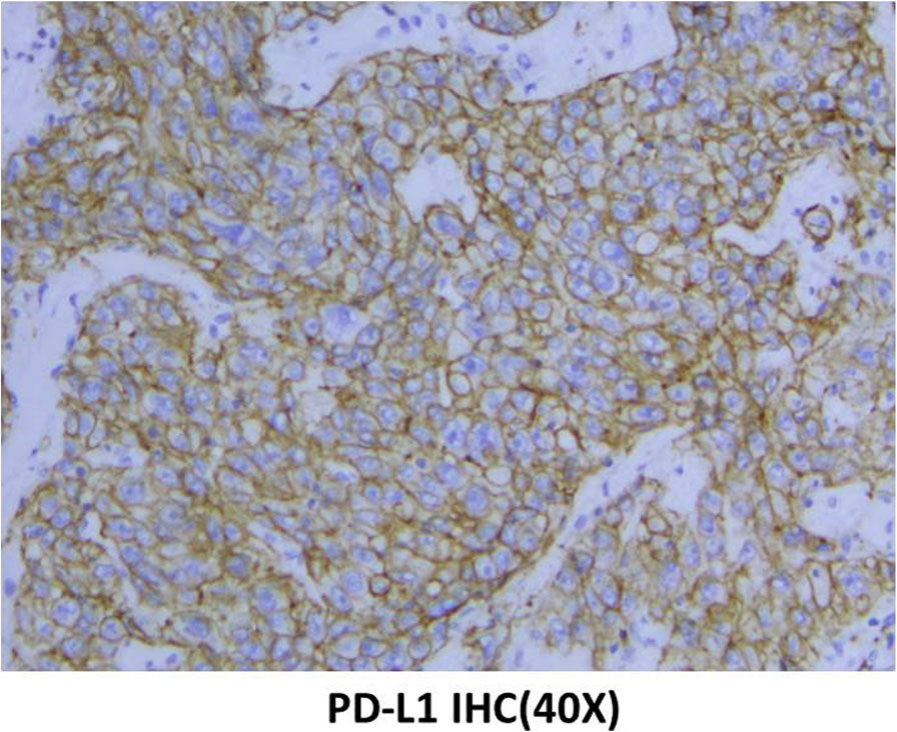
Immunohistochemical staining for PD-L1 expression (40X)

Two months after surgery, the patient underwent a computed tomography (CT) examination, which demonstrated a metastatic focus in the coelom (Fig. 2a and f). The patient received chemotherapy consisting of gemcitabine and cisplatin (GP). After four cycles, positron emission tomography-computed tomography (PET-CT) showed an increase in the number of metastatic lesions in the coelom, which suggested disease progression (Fig. 2b and g). With the patient’s consent, the tissue sample obtained during surgery was submitted for genomic alteration testing using a 450-gene panel as part of next generation sequencing (NGS); the tissue was also tested for the expression of PD-L1. Genomic testing showed the *TP53* R249S, *ATRX* K1177, and *RB1* K80 mutations, but no mutations were detected in the *ERBB2* or *KRAS* genes. The tumour mutational burden was 19.3 mut/Mb and was defined as TMB-high. The MSI status was stable. Immunohistochemistry (IHC) results showed that the PD-L1 TPS of the tissue sample was 80%, which indicated high expression of PD-L1 (Fig. 3). Moreover, MMR proteins were positive. With the patient^’^s consent, a combination therapy consisting of immunotherapy and chemotherapy was administered based on these findings. After treatment with pembrolizumab (150 mg q3w), oxaliplatin and tegafur [SOX] (oxaliplatin 130 mg/m2, d1, tegafur 60 mg BID, d1–14, q3w) for 4 cycles, CT showed that the lesions in the coelom had significantly decreased in size (Fig. 2c and h). Subsequently, the patient received combination therapy with pembrolizumab and SOX for another 3 cycles. During the 5th cycle of combined therapy, the patient experienced third-degree neutropenia with fever. Considering the side effects of the chemotherapy, the situation was improved after G-CSF treatment was administered. Then, the dose of chemotherapy was lowered to 80%. The lesions in the coelom had nearly resolved on the CT scans in July 2017 (Fig. 2d and i), which suggested that the disease was in complete remission. The patient received a total of 8 cycles of pembrolizumab+SOX and then received pembrolizumab maintenance monotherapy for 6 months. The drug treatment was stopped in February 2018 due to personal reasons. From July 2017 to May 2018, when was the patient was last followed-up, the cancer was in complete remission (CR) (Fig. 2e and j), and the toxicity associated with immunotherapy was not obvious.

## Discussion

Intrahepatic cholangiocarcinoma is a clinically challenging malignancy with a poor prognosis. Nearly no patients survive > 3 years without surgical therapy. Indeed, even after surgical resection, the 3-year survival rate is 40 to 50% [17]. Compared with other hepatobiliary cancers, iCCA is associated with decreased survival, a low resection rate, and a low cure rate. It is therefore imperative to explore effective therapies for iCCA. Studies have shown abnormal activation of several oncogenes and signalling pathways among patients diagnosed with iCCA. For example, *TP53* has been reported to be associated with poor outcome in patients with iCCA. *BRAF* mutations were shown to occur in approximately 5% of iCCA cases, and previous studies have shown that combination therapy with dabrafenib and trametinib were effective in iCCA patients with the BRAF V600E mutation [18, 19]. Approximately 11% of iCCAs were demonstrated to harbour gene fusions of FGFR1/2/3, which is rare in other cholangiocarcinomas. At present, BGJ398, a small molecule inhibitor of FGFR1–3, has also demonstrated a promising therapeutic effect [20].

The results of NGS revealed that the patient described here had a relatively high tumour mutational burden, which is closely related to the effects of immunotherapy. The Checkmate-026 clinical study has shown that nivolumab, compared with chemotherapy, does not lengthen the progression-free survival (PFS) of patients with NSCLC with positive PD-L1 expression. A retrospective analysis suggested that patients with a high TMB had significantly prolonged PFS when treated with nivolumab [21]. Moreover, patients with glioblastomas, endometrial carcinomas, or colorectal cancers with a high TMB are sensitive to immunotherapy with PD-1/PD-L1 inhibitors [22–24].

In the present case, immunohistochemical analysis revealed high expression of PD-L1 in the tumour tissue, with a positive rate as high as 80%. PD-1, an immune checkpoint inhibitor that has been reported to be associated with PD-L1 expression, has emerged as a potential therapeutic target in multiple cancers. Studies that involve the expression of PD-L1 in cholangiocarcinomas have been preliminary and not comprehensive. Specifically, in the KEYNOTE028 study, 23 patients with cholangiocarcinomas were treated with pembrolizumab alone; 4 patients achieved partial remission and 4 patients had stable disease. These results suggested that immunotherapy may be a potential therapy for cholangiocarcinoma [25]. Current findings on PD-1/PD-L1 showed that monotherapy was related to a low response rate (10 to 20%) [26], although some patients had stable disease. Infiltration of lymphocytes into the tumour microenvironment has been found to be related to the response rate and effect of PD-1/PD-L1 inhibitors. Chemotherapy can influence the tumour microenvironment, which leads to an increased infiltration of lymphocytes [27, 28]. All these signs allow the possibility of immunotherapy. Combination therapy is more effective than immunotherapy alone and can increase the effective response rate. Currently, the combination of immunotherapy and chemotherapy in many cancers has yielded high effective response rates and high efficacy [29]. KEYNOTE-021, a clinical trial involving 123 patients with NSCLC who were treated without chemotherapy, reported a remission rate of 55% and disease control rate of 88% after treatment with pembrolizumab and chemotherapy. The remission rate of patients treated with combination therapy was twice that of those treated with chemotherapy alone (29%) [30]. KEYNOTE-189 demonstrated that the addition of pembrolizumab to standard chemotherapy of pemetrexed and a platinum-based drug resulted in significantly longer overall survival and progression-free survival compared with chemotherapy alone in non-squamous NSCLC [31]. Considering the related research and our case, immunotherapy combined with chemotherapy may play a potential role in cholangiocarcinoma treatment.

## Conclusion

Here, we reported a Chinese patient with intrahepatic cholangiocarcinoma, a high TMB and high expression of PD-L1 who responded well to the combined therapy of pembrolizumab and chemotherapy. Targeted next-generation sequencing of hundreds of cancer genes in a panel played a key role in guiding therapy for this patient and may result in promising combination therapy options for advanced cholangiocarcinoma.

## References

[1] Flejou JF (2011). WHO classification of digestive tumors: the fourth edition. Ann Pathol.

[2] Yamamoto M, Takasaki K, Yoshikawa T (1999). Lymph node metastasis in intrahepatic cholangiocarcinoma. Jpn J Clin Oncol.

[3] National Comprehensive Cancer Network. Hepatobiliary cancers (Version 2.2018). 2018. Accessed 7 June 2018.

[4] Farley DR, Weaver AL, Nagorney DM (1995). “Natural history” of unresected cholangiocarcinoma: patient outcome after noncurative intervention. Mayo Clin Proc.

[5] Shaib YH, El-Serag HB, Davila JA, Morgan R, McGlynn KA (2005). Risk factors of intrahepatic cholangiocarcinoma in the United States: a case-control study. Gastroenterology.

[6] Rijnders M, de Wit R, Boormans JL, Lolkema MPJ, van der Veldt AAM (2017). Systematic review of immune checkpoint inhibition in urological cancers. Eur Urol.

[7] Lehman JM, Gwin ME, Massion PP (2017). Immunotherapy and targeted therapy for small cell lung Cancer: there is Hope. Curr Oncol Rep.

[8] Emens LA, Ascierto PA, Darcy PK, Demaria S, Eggermont AMM, Redmond WL, Seliger B, Marincola FM (2017). Cancer immunotherapy: opportunities and challenges in the rapidly evolving clinical landscape. Eur J Cancer.

[9] Herbst RS, Baas P, Kim DW, Felip E, Perez-Gracia JL, Han JY, Molina J, Kim JH, Arvis CD, Ahn MJ (2016). Pembrolizumab versus docetaxel for previously treated, PD-L1-positive, advanced non-small-cell lung cancer (KEYNOTE-010): a randomised controlled trial. Lancet.

[10] Ribas A, Hamid O, Daud A, Hodi FS, Wolchok JD, Kefford R, Joshua AM, Patnaik A, Hwu WJ, Weber JS (2016). Association of Pembrolizumab with Tumor Response and Survival among Patients with Advanced Melanoma. JAMA.

[11] Overman MJ, Lonardi S, Wong KYM, Lenz HJ, Gelsomino F, Aglietta M, Morse MA, Van Cutsem E, McDermott R, Hill A (2018). Durable clinical benefit with Nivolumab plus Ipilimumab in DNA mismatch repair-deficient/microsatellite instability-high metastatic colorectal Cancer. J Clin Oncol.

[12] Motzer RJ, Tannir NM, McDermott DF, Aren Frontera O, Melichar B, Choueiri TK, Plimack ER, Barthelemy P, Porta C, George S (2018). Nivolumab plus Ipilimumab versus Sunitinib in advanced renal-cell carcinoma. N Engl J Med.

[13] Viale G, Trapani D, Curigliano G (2017). Mismatch repair deficiency as a predictive biomarker for immunotherapy efficacy. Biomed Res Int.

[14] Maleki Vareki S, Garrigos C, Duran I (2017). Biomarkers of response to PD-1/PD-L1 inhibition. Crit Rev Oncol Hematol.

[15] Chalmers ZR, Connelly CF, Fabrizio D, Gay L, Ali SM, Ennis R, Schrock A, Campbell B, Shlien A, Chmielecki J (2017). Analysis of 100,000 human cancer genomes reveals the landscape of tumor mutational burden. Genome Med.

[16] Reck M, Rodriguez-Abreu D, Robinson AG, Hui R, Csoszi T, Fulop A, Gottfried M, Peled N, Tafreshi A, Cuffe S (2016). Pembrolizumab versus chemotherapy for PD-L1-positive non-small-cell lung cancer. N Engl J Med.

[17] Dodson RM, Weiss MJ, Cosgrove D, Herman JM, Kamel I, Anders R, Geschwind JF, Pawlik TM (2013). Intrahepatic cholangiocarcinoma: management options and emerging therapies. J Am Coll Surg.

[18] Lavingia V, Fakih M (2016). Impressive response to dual BRAF and MEK inhibition in patients with BRAF mutant intrahepatic cholangiocarcinoma-2 case reports and a brief review. J Gastrointest Oncol.

[19] Loaiza-Bonilla A, Clayton E, Furth E, O’Hara M, Morrissette J (2014). Dramatic response to dabrafenib and trametinib combination in a BRAF V600E-mutated cholangiocarcinoma: implementation of a molecular tumour board and next-generation sequencing for personalized medicine. Ecancermedicalscience.

[20] Nogova L, Sequist LV, Perez Garcia JM, Andre F, Delord JP, Hidalgo M, Schellens JH, Cassier PA, Camidge DR, Schuler M (2017). Evaluation of BGJ398, a fibroblast growth factor receptor 1-3 kinase inhibitor, in patients with advanced solid tumors harboring genetic alterations in fibroblast growth factor receptors: results of a global phase I, dose-escalation and dose-expansion study. J Clin Oncol.

[21] Carbone DP, Reck M, Paz-Ares L, Creelan B, Horn L, Steins M, Felip E, van den Heuvel MM, Ciuleanu TE, Badin F (2017). First-line Nivolumab in stage IV or recurrent non-small-cell lung Cancer. N Engl J Med.

[22] Hodges TR, Ott M, Xiu J, Gatalica Z, Swensen J, Zhou S, Huse JT, de Groot J, Li S, Overwijk WW (2017). Mutational burden, immune checkpoint expression, and mismatch repair in glioma: implications for immune checkpoint immunotherapy. Neuro-Oncology.

[23] Ahn SM, Ansari AA, Kim J, Kim D, Chun SM, Kim J, Kim TW, Park I, Yu CS, Jang SJ (2016). The somatic POLE P286R mutation defines a unique subclass of colorectal cancer featuring hypermutation, representing a potential genomic biomarker for immunotherapy. Oncotarget.

[24] Gargiulo P, Della Pepa C, Berardi S, Califano D, Scala S, Buonaguro L, Ciliberto G, Brauchli P, Pignata S (2016). Tumor genotype and immune microenvironment in POLE-ultramutated and MSI-hypermutated endometrial cancers: new candidates for checkpoint blockade immunotherapy?. Cancer Treat Rev.

[25] Bang Y.J., Doi T., Braud F. De, Piha-Paul S., Hollebecque A., Razak A.R. Abdul, Lin C.C., Ott P.A., He A.R., Yuan S.S., Koshiji M., Lam B., Aggarwal R. (2015). 525 Safety and efficacy of pembrolizumab (MK-3475) in patients (pts) with advanced biliary tract cancer: Interim results of KEYNOTE-028. European Journal of Cancer.

[26] Iwai Y, Hamanishi J, Chamoto K, Honjo T (2017). Cancer immunotherapies targeting the PD-1 signaling pathway. J Biomed Sci.

[27] Takakura H, Domae S, Ono T, Sasaki A (2017). The immunological impact of chemotherapy on the tumor microenvironment of Oral squamous cell carcinoma. Acta Med Okayama.

[28] Sato Y, Gonda K, Harada M, Tanisaka Y, Arai S, Mashimo Y, Iwano H, Sato H, Ryozawa S, Takahashi T (2017). Increased neutrophil-to-lymphocyte ratio is a novel marker for nutrition, inflammation and chemotherapy outcome in patients with locally advanced and metastatic esophageal squamous cell carcinoma. Biomed Rep.

[29] Gotwals P, Cameron S, Cipolletta D, Cremasco V, Crystal A, Hewes B, Mueller B, Quaratino S, Sabatos-Peyton C, Petruzzelli L (2017). Prospects for combining targeted and conventional cancer therapy with immunotherapy. Nat Rev Cancer.

[30] Langer CJ, Gadgeel SM, Borghaei H, Papadimitrakopoulou VA, Patnaik A, Powell SF, Gentzler RD, Martins RG, Stevenson JP, Jalal SI (2016). Carboplatin and pemetrexed with or without pembrolizumab for advanced, non-squamous non-small-cell lung cancer: a randomised, phase 2 cohort of the open-label KEYNOTE-021 study. Lancet Oncol.

[31] Gandhi L, Rodriguez-Abreu D, Gadgeel S, Esteban E, Felip E, De Angelis F, Domine M, Clingan P, Hochmair MJ, Powell SF (2018). Pembrolizumab plus chemotherapy in metastatic non-small-cell lung cancer. N Engl J Med.

